# Influence of Dietary Fermented Coffee Cherry Pulp on Growth Performance, Meat Quality, and Cecal Microbiota in Thai Native Chickens

**DOI:** 10.3390/ani16060965

**Published:** 2026-03-19

**Authors:** Orranee Srinual, Phatchari Srinual, Krit Khetanun, Pong Loungmoon, Naret Pintalerd, Thanongsak Chaiyaso, Kamon Yakul, Chanidapha Kanmanee, Wanaporn Tapingkae

**Affiliations:** 1 Department of Animal and Aquatic Sciences, Faculty of Agriculture, Chiang Mai University, Chiang Mai 50200, Thailand; orranee.s@cmu.ac.th (O.S.); phatchari_sri@cmu.ac.th (P.S.); chanidapha.k@cmu.ac.th (C.K.); 2 Functional Feed Innovation Center (FuncFeed), Faculty of Agriculture, Chiang Mai University, Chiang Mai 50200, Thailand; 3 Highland Research and Training Center, Faculty of Agriculture, Chiang Mai University, Chiang Mai 50200, Thailand; krit.khet@cmu.ac.th (K.K.); pong.loungmoon@cmu.ac.th (P.L.); naret.p@cmu.ac.th (N.P.); 4 Division of Biotechnology, Faculty of Agro-Industry, Chiang Mai University, Chiang Mai 50100, Thailand; thanongsak.c@cmu.ac.th (T.C.); kamon.y@cmu.ac.th (K.Y.); 5 Office of Research Administration, Chiang Mai University, Chiang Mai 50200, Thailand

**Keywords:** fermented coffee cherry pulp, antibiotic growth promoter alternative, gut microbiota modulation, Thai native chickens

## Abstract

Coffee cherry pulp is an abundant by-product of coffee processing and contains bioactive compounds with potential value in animal nutrition. This study evaluated fermented coffee cherry pulp (CCF) as a feed additive in Thai native chickens. Five hundred day-old chicks were randomly allocated to five treatments: basal diet (CON), antibiotic growth promoter (AGP), and basal diets supplemented with 0.5, 1.0, or 2.0 g/kg CCF for 12 weeks. Supplementation with 1.0 g/kg CCF significantly increased final body weight and average daily gain and significantly improved feed conversion ratio compared with the basal diet (*p* < 0.05). Serum triglyceride levels were significantly reduced in the CCF1.0 and CCF2.0 groups (*p* < 0.05), while liver and kidney function indicators were not affected. Cooking loss was significantly lower in CCF-supplemented groups, and thigh muscle crude protein content was significantly higher at 0.5 and 1.0 g/kg (*p* < 0.05). Alpha diversity of cecal microbiota was not significantly altered, but directional shifts in community composition were observed, including enrichment of short-chain fatty acid-associated genera. Functional prediction indicated differences in carbohydrate and amino acid metabolic pathways. These results support fermented coffee cherry pulp as a potential alternative to antibiotic growth promoters in native chicken production.

## Simple Summary

Coffee processing generates large amounts of by-products that are often discarded despite containing valuable bioactive compounds. This study evaluated fermented coffee cherry pulp (CCF) as a natural feed additive for slow-growing chickens. Supplementation at an optimal level improved growth performance, feed efficiency, blood lipid profile, and selected meat quality traits without negative effects on health. Although overall gut microbial diversity remained stable, specific beneficial bacterial groups associated with fermentation processes were enriched. These findings indicate that CCF can enhance production efficiency while supporting gut health, offering a sustainable and natural alternative to antibiotic growth promoters in poultry production.

## 1. Introduction

The global coffee industry generates substantial quantities of agricultural by-products, most of which end up as waste and create environmental problems [1,2]. It has been estimated that coffee production generates over 2 billion tons of solid waste worldwide, including residues such as coffee pulp, husks, silverskin, and spent coffee grounds, which may pose environmental challenges if not properly managed [3]. Coffee cherry pulp (CCP), which comprises the outer skin and mucilage removed during processing, is one such by-product [4]. Although traditionally considered waste, CCP is rich in carbohydrates, fiber, phenolic compounds, and other bioactive constituents. Its richness in polyphenols and antioxidants highlights CCP as a promising bioresource for value-added use [5,6].

One promising approach to valorize CCP is microbial fermentation, which can enhance antioxidative compounds and improve nutrient bioavailability [7]. Yeasts are effective in enhancing and bioactive profile of various agricultural by-products, particularly CCP [8]. In this study, we focus on *Kluyveromyces marxianus* ST5, a pectinolytic yeast known for its exceptional pectin-degrading enzyme production and generally recognized as safe (GRAS), making it suitable for food industry applications [9,10]. Moreover, *Kluyveromyces marxianus* ST5 demonstrates robust pectinolytic activity, thermotolerance, rapid growth, and broad substrate utilization, making it an ideal candidate for the agro-industrial bioconversion of agricultural biomass into high-value products [9].

Fermentation of Arabica coffee cherry pulp alters its chemical composition and sensory characteristics. Fermentation can transform complex compounds in CCP into simpler molecules with enhanced bioactivity. This process not only increases the total polyphenol and flavonoid content but also enhances the antioxidant activity of CCP [11]. In addition, using pectinolytic microorganisms during fermentation can reduce antinutritional compounds present in raw CCP, including tannins and phytates, thereby improving its safety and suitability as a functional feed supplement for animal nutrition [10].

CCP has gained increasing attention as a potential feed ingredient because of its rich antioxidant profile and the presence of other bioactive compounds [1]. These components are thought to support growth performance, contribute to maintaining gut microbial balance and reduce the proliferation of pathogenic bacteria [12]. Key constituents, including caffeic acid, melanoidins, and chlorogenic acid, are considered to play an important role in these potential benefits [13,14], positioning CCP as a promising value-added by-product in animal nutrition.

Natural feed additives are increasingly investigated as substitutes for antibiotic growth promoters in poultry production. Nevertheless, in Thailand, antibiotic growth promoters remain commonly used in small-scale farming systems, particularly in Thai native chicken production. Although fermentation of coffee cherry pulp with *Kluyveromyces marxianus* ST5 has been reported to enhance antioxidant capacity under in vitro conditions [10], information regarding its in vivo effects in Thai native chickens remains limited. To our knowledge, this is one of the first studies to investigate the effects of fermented coffee cherry pulp on growth performance, meat quality, and cecal microbiota in Thai native chickens. Therefore, this study aimed to evaluate the effects of fermented coffee cherry pulp (CCF) supplementation on growth performance, serum biochemical parameters, meat quality, cecal microbiota composition, and predicted microbial functional profiles in Thai native chickens.

## 2. Materials and Methods

### 2.1. Preparation of Fermented Coffee Cherry Pulp as a Feed Additive

Coffee cherry pulp (CCP; *Coffea arabica* L.) was obtained from Highland Research and Training Center, Faculty of Agriculture, Chiang Mai University, Chiang Mai, Thailand. The fresh pulp was oven-dried at 60 °C for 48 h and subsequently ground to obtain dried CCP. For fermentation, the dried CCP was used as the primary substrate following the protocol described by Chaiyaso et al. [10] with minor modifications. Briefly, CCP was prepared at the desired concentration 16.81 (*w*/*v*) in distilled water supplemented with inorganic nitrogen sources and minerals. The initial pH was adjusted to 5.2–5.5 using 6 N NaOH before sterilization. The medium was autoclaved at 121 °C for 15 min. A starter culture of *Kluyveromyces marxianus* ST5 was prepared in yeast malt (YM) broth and incubated at 30 °C for 24–48 h under agitation. The fermentation medium was inoculated with 5.87% (*v*/*v*) of the actively growing yeast culture and incubated at 30 °C for 48 h under static submerged fermentation conditions.

After fermentation, the whole fermented CCP was collected and freeze-dried (Labconco, FreezeZone 6, Kansas City, MO, USA) to obtain a dry fermented CCP powder. The dried product was ground and sieved to a uniform particle size (approximately 0.1 mm) to ensure homogeneity. The final fermented CCP powder was stored at 4 °C in airtight containers until further use as a functional feed additive. The bioactive compound profile and antioxidant capacity demonstrated in this study are in agreement with those previously reported by Chaiyaso et al. [10], with notable enhancement in antioxidant activity. These results substantiate its potential as a functional feed additive.

### 2.2. Animal, Diet, and Experimental Design

This study was approved by the Department of Animal and Aquatic Animal Sciences at the Faculty of Agriculture, Chiang Mai University, Thailand. All experimental procedures were following the guidelines established by the Animal Ethics Committee, Faculty of Agriculture, Chiang Mai University. The ethical approval code for this study was RAGIACUC034/2567. A total of 500 one-day-old Thai native crossbred chickens (Pradu-Hangdum) with similar initial body weights of 33.9 ± 4.0 g were randomly allocated to five treatment groups in a completely randomized design, with ten replicate pens per treatment and ten birds per pen. The experimental groups were defined as follows: one group received a basal diet without additives (CON), whereas another group was administered the basal diet supplemented with an antibiotic (AGP), which consisted of a mixture of amoxicillin (100 g/kg) and colistin (400 × 10^6^ IU/kg) at a ratio of 1.0 g/kg. Additionally, three groups were supplemented with 0.5, 1.0, and 2.0 g/kg of coffee cherry pulp fermented (CCF) for 35 days. The selected inclusion levels were based on the phenolic-rich nature of fermented coffee cherry pulp, particularly its chlorogenic acid- and caffeic acid-associated antioxidant potential, together with our previous in vitro findings showing improved antioxidant activity and digestibility after fermentation [10]. The range of 0.5–2.0 g/kg diet was further chosen to match practically relevant supplementation levels reported for coffee-pulp-derived additives in poultry, allowing evaluation of biological responses without excessive inclusion of the test material. Table 1 presents the ingredients of the basal diets. All chickens were raised in cages (150 cm [length] × 70 cm [width] × 50 cm [height]). During the trial, all birds were housed in the same environmentally controlled poultry house with *ad libitum* access to feed and water. The initial temperature was maintained at 32 °C and gradually reduced by 2–3 °C per week until reaching 25 ± 2 °C, with relative humidity maintained at approximately 70 ± 5%. A lighting program of 18 h light and 6 h dark was applied throughout the experimental period. Birds were vaccinated according to the standard vaccination recommendations of the Department of Livestock Development of Thailand, including Newcastle disease (ND) and infectious bronchitis (IB) vaccines administered between 1 and 7 days of age, followed by the infectious bursal disease (Gumboro) vaccine administered between 7 and 14 days of age. As part of the routine farm vaccination program, booster vaccinations against Newcastle disease, infectious bronchitis, and fowl cholera were administered every three months.

### 2.3. Growth Performance Measurements

At the beginning of the experiment and subsequently each week thereafter, the weight of each individual chicken was recorded. Weekly feed intake and body weight (BW) for each experimental unit were monitored, allowing for the calculation of key performance indicators, including average daily feed intake (ADFI), average daily gain (ADG) and feed conversion ratio (FCR). Feed intake (FI) was calculated by subtracting the amount of feed remaining from the amount of feed provided on each feed day. ADG = (finale weight − initial weight)/(day on feed × number of broilers); ADFI = feed intake/(day on feed × number of chickens); FCR = Total feed intake/weight gain [15].

### 2.4. Serum Biochemistry and Lipid Profiles

Blood was collected from ten chickens from each group at the age of 35 days of the experiment. Blood samples were obtained from the wing vein and were transferred into sterile tubes devoid of anticoagulants. The serum was separated via centrifugation at 3000 rpm for 15 min and subsequently stored at −20 °C in a refrigerator for subsequent analysis [16]. Serum biochemical indices related to renal function, hepatic enzymes, and protein metabolism were evaluated, including blood urea nitrogen, creatinine, AST, ALT, ALP, total protein, albumin, and globulin. In addition, lipid profile parameters (HDL, LDL, total cholesterol, and triglycerides) were measured. The methodologies employed for the analysis of both lipid profiles and serum biochemistry followed the guidelines established by Ding et al. [16] and Ashour et al. [17]. Analyses were conducted using commercially available kits (DiaSys Diagnostic Systems GmbH; Holzheim, Germany), and the results were processed with the Automated Chemistry Analyzer (BX-3010, Sysmex Corporation of Japan, Kobe, Japan).

### 2.5. Meat Quality

After slaughter, the breasts were immediately packed individually in sealable plastic bags and stored at 4 °C for the measurement of meat quality [16]. Ten samples per treatment were prepared for meat quality determination. The pH (45 min and 24 h postmortem), drip loss (24 h), and meat color (L*, a*, b*) were measured as previously described [12]. Cooking loss and shear force were determined using an Instron Universal Testing Machine (Model 3433, Norwood, MA, USA) according to the referenced protocol [12,15]. For proximate composition analysis, breast and thigh muscle samples (*n* = 10 per treatment) were homogenized and analyzed for dry matter (%DM), moisture (%Moisture), ash (%Ash), ether extract (%EE), and crude protein (%CP) according to AOAC [18]. All analyses were performed in triplicate and expressed on a percentage basis.

### 2.6. Cecal Digesta Collection and Microbial DNA Extraction

At the end of the 12-week feeding trial, one bird per pen (*n* = 10 per treatment) was randomly selected and humanely euthanized by cervical dislocation in accordance with institutional animal care guidelines. Immediately after slaughter, the abdominal cavity was aseptically opened, and the ceca were carefully excised. Approximately 1 g of cecal digesta was collected into sterile cryovials under aseptic conditions. Samples were immediately snap-frozen in a portable −20 °C freezer during transport and subsequently stored at −80 °C until further analysis.

Microbial genomic DNA was extracted from 220 mg of homogenized cecal digesta using the QIAamp Fast DNA Stool Mini Kit (Qiagen, Hilden, Germany) following the manufacturer’s instructions. DNA quantity was determined using a Qubit 2.0 fluorometer (Thermo Fisher Scientific, Waltham, MA, USA), and purity was assessed by spectrophotometry (A260/A280 ratio) using a spectrophotometer (Thermo Fisher Scientific, Waltham, MA, USA). DNA integrity was verified by 1% agarose gel electrophoresis. Only samples with A260/A280 ratios between 1.8 and 2.0 and clear intact bands were used for downstream sequencing. Extracted DNA was stored at −80 °C until PCR amplification [19].

### 2.7. 16S rRNA Gene Amplification and Sequencing

The V3–V4 hypervariable region of the bacterial 16S rRNA gene was amplified using universal primers 341F (5′-CCTAYGGGRBGCASCAG-3′) and 806R (5′-GGACTACNNGGGTATCTAAT-3′) [20]. PCR reactions were performed using Herculase II Fusion DNA Polymerase (Agilent Technologies, Santa Clara, CA, USA) under the following conditions: initial denaturation at 94 °C for 3 min; 35 cycles of denaturation at 94 °C for 45 s, annealing at 50 °C for 60 s, extension at 72 °C for 10 min; and final extension at 72 °C for 5 min [20]. PCR products were verified by 2% agarose gel electrophoresis and purified using AMPure XP magnetic beads (Beckman Coulter, Indianapolis, IN, USA). Sequencing libraries were constructed according to standard Illumina protocols and sequenced on the Illumina MiSeq platform (Illumina, San Diego, CA, USA) using paired-end 2 × 250 bp chemistry [21].

### 2.8. Bioinformatic Processing and Microbial Community Analysis

Raw paired-end reads were processed using QIIME2 (version 2023.5). Low-quality reads and chimeric sequences were removed, and denoising was performed using the DADA2 algorithm to generate amplicon sequence variants (ASVs) [22]. Taxonomic classification was conducted against the SILVA 138 reference database. Taxonomic classification was assigned using a pre-trained Naïve Bayes classifier against the SILVA reference database (release 138). Non-bacterial sequences (e.g., chloroplast and mitochondrial reads) were removed before downstream analysis. Feature tables and taxonomic abundance tables at the phylum, family, and genus levels were exported for diversity and compositional analyses.

#### 2.8.1. Alpha and Beta Diversity

Alpha diversity indices (Chao1 and Shannon evenness) were calculated in QIIME2. Differences among treatments were assessed using the Wilcoxon rank-sum test. Beta diversity was evaluated using Bray–Curtis dissimilarity and visualized through Principal Coordinates Analysis (PCoA). Community structure differences were tested using PERMANOVA (Adonis function in vegan package, R software version 4.4.3), and the homogeneity of dispersion was assessed using PERMDISP (betadisper function in the vegan package, R version 4.4.3) [23].

#### 2.8.2. Taxonomic Composition Analysis

Relative abundance of bacterial taxa was calculated at the phylum, family, and genus levels. Taxonomic composition was visualized using stacked bar plots. To evaluate differences in taxonomic distribution among treatments, a heatmap was generated based on predominant taxa. Relative abundance values were standardized using Z-score transformation to facilitate comparison across samples. Hierarchical clustering was performed using Euclidean distance and complete linkage to identify treatment-related grouping patterns.

#### 2.8.3. Functional Prediction of Microbial Communities

Functional potential of the cecal microbiota was inferred using PICRUSt2 based on 16S rRNA gene profiles. Predicted gene functions were mapped to the KEGG and MetaCyc databases. Only KEGG Level 2 pathways and dominant MetaCyc pathways were retained for downstream analysis. Differentially abundant functional pathways between dietary treatments were identified using DESeq2 with FDR correction (adjusted *p* < 0.05) [24].

### 2.9. Statistical Analysis

Growth performance, serum biochemistry, carcass characteristics and meat quality parameters were analyzed using SPSS version 22.0 (SPSS Inc., Chicago, IL, USA). Differences among treatments were evaluated using one-way analysis of variance (ANOVA). When significant effects were detected, treatment means were compared using Duncan’s multiple range test. For variables that did not meet the assumptions of normality or homogeneity of variance, the non-parametric Kruskal–Wallis test was applied. Statistical significance was declared at *p* < 0.05. Microbial community analyses were performed in R (v3.3.1). Alpha diversity was compared using the Wilcoxon rank-sum test. Beta diversity was calculated based on Bray–Curtis dissimilarity, visualized by principal coordinate analysis (PCoA), and evaluated using PERMANOVA. Differential taxa were identified using LEfSe (version 1.0). Spearman correlation analysis was conducted to assess associations between microbial taxa, predicted pathways, and serum parameters. Statistical significance was set at *p* < 0.05.

## 3. Results

### 3.1. Growth Performance

As shown in Table 2, dietary CCF supplementation had no significant effect on the initial body weight. However, significant differences were observed in final body weight, ADG, ADFI and FCR. Chickens fed 1.0 g/kg CCF showed the highest final weight and ADG but were not significantly different from AGP, whereas the control, CCF0.5, and CCF2.0 were lower (*p* < 0.01). The CCF1.0 group had the lowest ADFI compared to the other groups (*p* < 0.01). In terms of FCR, the CCF1.0 group exhibited the most efficient feed utilization with the lowest FCR (3.05), compared with CON (3.63), AGP (3.60), CCF0.5 (3.65), and CCF2.0 (3.59) (*p* < 0.01).

### 3.2. Serum Biochemical Parameters and Lipid Profile

As presented in Table 3, dietary CCF supplementation affected certain serum biochemical parameters and lipid profiles of Thai native chickens. BUN was significantly higher in the control group and lower in AGP and CCF0.5 groups; however, these values did not differ from CCF1.0 and CCF2.0. Creatinine, AST, ALT, total protein, albumin, total cholesterol, HDL, and LDL did not differ significantly among treatments (*p* > 0.05). Alkaline phosphatase (ALP) was significantly higher in the control (1062.80 U/L) and CCF1.0 (1136.67 U/L) groups than in AGP (821.33 U/L), CCF0.5 (821.33 U/L), and CCF2.0 (850.33 U/L) (*p* < 0.05). Moreover, globulin concentration was significantly higher in CCF2.0 4.47 g/dL) compared with other treatments (*p* < 0.05). For the lipid profile, triglyceride concentration was highest in the AGP group (45.33 mg/dL), did not differ from the control (39.00 mg/dL) or CCF0.5 (40.67 mg/dL), and was lower in CCF1.0 (30.67 mg/dL) and CCF2.0 (25.33 mg/dL) (*p* < 0.05).

### 3.3. Meat Quality

The effects of CCF on meat quality and muscle chemical composition are presented in Table 4. Dietary supplementation with CCF did not affect breast muscle pH at 45 min or 24 h postmortem nor the rate of pH decline (*p* > 0.05). However, breast meat lightness (L*) was significantly increased in CCF2.0 compared with other groups (*p* < 0.05).

In addition, cooking loss was significantly reduced in AGP and all CCF groups compared with CON (*p* < 0.001). Drip loss was not significantly affected (*p* > 0.05), although numerically lower values were observed in CCF1.0 and CCF2.0. Shear force did not differ among treatments (*p* > 0.05).

Moreover, significant differences were observed in the chemical composition of thigh muscle. Dry matter (%DM) was higher in the AGP, CCF0.5, and CCF1.0 groups compared with CON (*p* < 0.001), whereas moisture content showed the opposite trend. Notably, crude protein content in thigh muscle was significantly increased in CCF0.5 and CCF1.0 compared with CON and AGP groups (*p* < 0.05), with CCF2.0 showing intermediate values. In contrast, no significant differences were observed in the chemical composition of breast muscle among treatments (*p* > 0.05). These differences between muscle types may be related to their distinct physiological characteristics. In chickens, thigh muscle contains a higher proportion of oxidative fibers and exhibits greater metabolic activity, whereas breast muscle is predominantly composed of glycolytic fibers, which may make thigh muscle more responsive to dietary modulation of nutrient utilization and protein deposition.

### 3.4. Cecal Microbial Diversity and Structure

**Figure 1:**
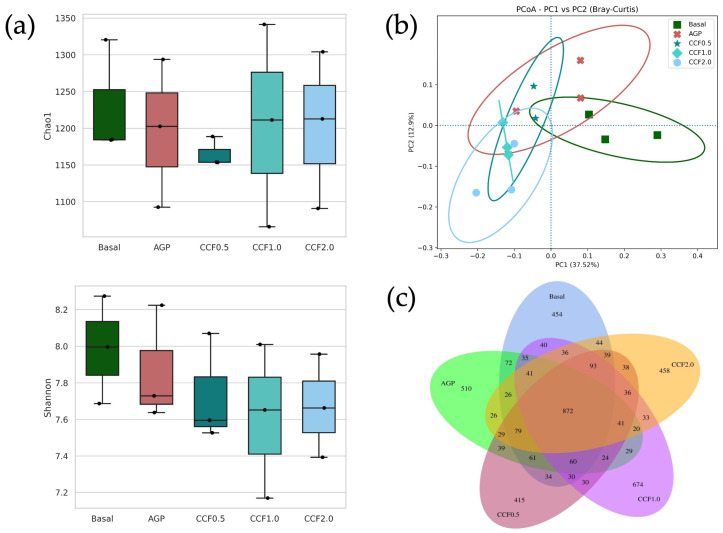
Effects of fermented coffee cherry pulp supplementation on cecal microbial in Thai native chickens. (**a**) Alpha diversity (Chao1 and Shannon indices). (**b**) PCoA based on Bray–Curtis dissimilarity (PC1 = 37.52%, PC2 = 12.9%). Ellipses indicate 95% confidence intervals. (**c**) Venn diagram illustrating shared and unique taxa among treatments. Basal: the control diet group; AGP: birds supplemented with antibiotic; CCF0.5, 1.0 and 2.0: diet supplemented with 0.5, 1.0 and 2.0 g/kg CCF.

Alpha diversity analysis (Figure 1a) indicated that dietary CCF supplementation did not significantly affect microbial richness or diversity, as reflected by comparable Chao1 and Shannon indices among treatments. These findings indicate that CCF inclusion maintained overall microbial diversity in the cecal microbiota.

However, Bray–Curtis-based PCoA indicated a directional shift in microbial community structure between CCF supplemented groups and the basal diet, with PC1 explaining 37.52% of the total variation. CCF treatments tended to cluster along the negative axis of PC1, whereas the basal diet group was positioned toward the positive axis. However, pairwise PERMANOVA did not reveal statistically significant differences among treatments (*p* > 0.05), although moderate effect sizes (R^2^ = 0.23–0.35) were observed (Figure 1b).

Venn analysis further (Figure 1a) revealed a substantial core microbiota shared among all groups (872 taxa), while CCF supplemented diets exhibited distinct sets of unique taxa, particularly in the CCF1.0 group. Differences in community structure were observed without significant changes in alpha diversity.

### 3.5. Taxonomic Composition of Cecal Microbiota

**Figure 2:**
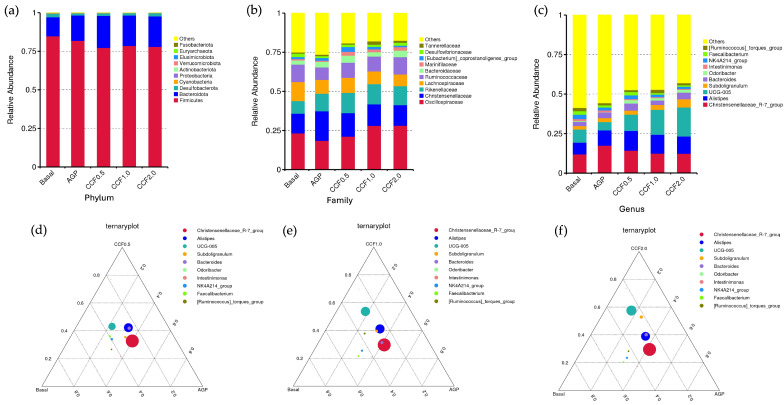
Taxonomic composition and directional distribution of the cecal microbiota in Thai native chickens. Relative abundance of dominant taxa at the (**a**) phylum, (**b**) family, and (**c**) genus levels (top 10 taxa). (**d**–**f**) Ternary plots showing the relative distribution of dominant genera among Basal, AGP, and CCF treatments at 0.5, 1.0, and 2.0 g/kg, respectively.

At the phylum level (Figure 2a), the cecal microbiota of Thai native chickens were predominantly composed of *Firmicutes*, followed by *Bacteroidota*, across all treatments. CCF supplementation did not significantly affect the microbial composition at the phylum level (*p* > 0.05). However, a slight reduction in *Firmicutes* and a corresponding increase in *Bacteroidota* were noted in the CCF groups compared with the basal diet group.

At the family level (Figure 2b), dominant taxa included *Oscillospiraceae*, *Christensenellaceae*, *Rikenellaceae*, *Lachnospiraceae* and *Ruminococcaceae*. CCF supplementation, particularly at 1.0% and 2.0%, was associated with an increased relative abundance of *Oscillospiraceae* and *Christensenellaceae*, whereas AGP showed a comparatively higher proportion of *Ruminococcaceae*.

At the genus level (Figure 2c), key genera included *Christensenellaceae*_R-7_group, *Alistipes*, UCG-005, *Bacteroides* and *Subdoligranulum*. Notably, CCF treatments exhibited a gradual increase in UCG-005 and *Subdoligranulum* with increasing inclusion levels. Overall, relative abundances of specific taxa differed among treatments. To further visualize treatment-related distribution patterns, ternary plots were constructed (Figure 2d–f). These plots illustrated a progressive shift of UCG-005 and *Subdoligranulum* toward the CCF vertex with increasing inclusion levels, whereas *Christensenellaceae*_R-7_group and *Alistipes* were more closely associated with AGP supplementation. Collectively, these findings indicate that CCF supplementation induced a directional compositional modulation distinct from antibiotic treatment, despite the overall conservation of core microbiota structure.

### 3.6. Heatmap and Hierarchical Clustering of Cecal Microbial Composition

**Figure 3:**
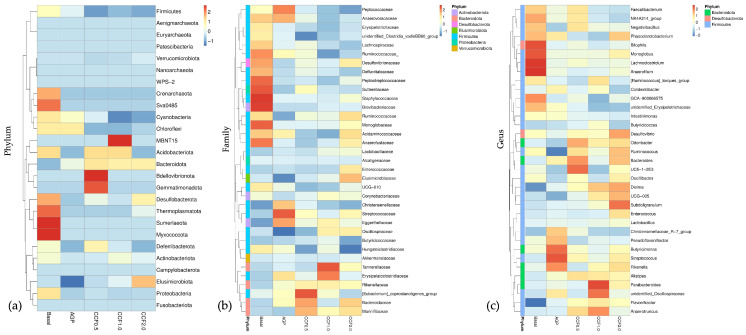
Heatmap analysis of differential microbial taxa in cecal microbiota of Thai native chickens. Heatmaps show relative abundance of predominant taxa at the (**a**) phylum, (**b**) family, and (**c**) genus levels. Colors represent row-wise standardized abundance (Z-score).

Heatmap analysis (Figure 3) indicated that the overall microbial structure remained stable across treatments, with subtle, treatment-dependent shifts in specific taxa. Hierarchical clustering showed partial grouping of CCF-supplemented treatments.

Firmicutes remained dominant at the phylum level across all groups, with minor variation in *Bacteroidota*. At the family and genus levels, key taxa including *Oscillospiraceae*, *Christensenellaceae*, *Alistipes*, UCG-005, and *Subdoligranulum* displayed treatment-related variation, particularly in the CCF1.0 and CCF2.0 groups. Overall, CCF supplementation modulated selected bacterial populations without disrupting the core microbiota.

### 3.7. PICRUSt Functional Prediction Analysis

**Figure 4:**
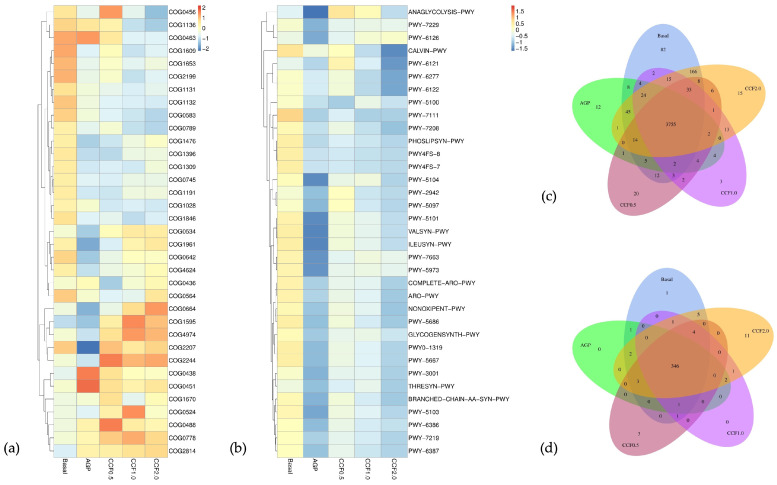
Predicted functional profiles of cecal microbiota in Thai native chickens across treatments. (**a**) Heatmap of differentially abundant COG functions. (**b**) Heatmap of MetaCyc pathway profiles. (**c**) Venn diagram of shared and unique COG functions among treatment. (**d**) Venn diagram of shared and unique MetaCyc pathways among treatment. Color scale indicates row-wise standardized abundance (Z-score).

Cluster of Orthologous Groups (COG)-based functional profiling (Figure 4a) showed that the core functional structure of the cecal microbiota was largely conserved across treatments, as reflected by the substantial overlap of shared COG categories (Figure 4c). However, relative abundances of specific COG categories differed between CCF supplemented groups and the Basal and AGP groups. Functional Prediction (Supplementary Tables S1 and S2) refers to the inference of microbial functional potential based on PICRUSt analysis, including predicted relative abundances and annotations of Cluster of Orthologous Groups (COG) categories (Table S1) and MetaCyc metabolic pathways (Table S2) across dietary treatments.

CCF at 1.0% and 2.0% exhibited higher relative abundances of regulatory and signal transduction-related functions than AGP and were comparable to or slightly elevated relative to Basal. Transcriptional regulator families, including LysR (COG0583), XRE-family regulators (COG1476), and MarR family proteins (COG1846), together with histidine kinases (COG0642) and GGDEF-domain proteins (COG2199), showed higher relative abundances in CCF1.0 and CCF2.0 compared with AGP. Several COG categories showed higher relative abundances in CCF1.0 and CCF2.0 compared with AGP.

Membrane-associated and transport functions were also more prominent in CCF groups compared with AGP. ABC-type lipoprotein export systems (COG1136), O-antigen and teichoic acid export proteins (COG2244), and MFS-family efflux permeases (COG2814) displayed numerically higher abundances in CCF1.0 and CCF2.0 relative to AGP and were comparable to or slightly higher than Basal.

These metabolic enzymes showed higher relative abundance in CCF groups compared with AGP. Ribokinase family enzymes (COG0524), NAD(P)-dependent dehydrogenases (COG1028), and iron-only hydrogenases (COG4624) were moderately enriched in CCF1.0 and CCF2.0 compared with AGP, while remaining similar to or slightly above Basal levels.

At the pathway level (Figure 4b), CCF1.0 demonstrated modest increases in central carbohydrate metabolism compared with AGP and CCF0.5, including Glycolysis III (ANAGLYCOLYSIS-PWY) and the non-oxidative pentose phosphate pathway (NONOXIPENT-PWY). Fermentation to acetate and lactate (PWY-5100), which was reduced in CCF0.5, was partially restored in CCF1.0 and CCF2.0 to levels approaching Basal and exceeding AGP.

Amino acid biosynthesis pathways, particularly branched-chain amino acid synthesis (BRANCHED-CHAIN-AA-SYN-PWY; ILEUSYN-PWY; VALSYN-PWY), were numerically higher in CCF1.0 and CCF2.0 than in AGP and CCF0.5 and were comparable to or slightly above Basal. Similarly, lipid biosynthesis pathways, including phospholipid biosynthesis (PHOSLIPSYN-PWY) and CDP-diacylglycerol biosynthesis (PWY-5667, PWY0-1319), were modestly elevated in CCF1.0 relative to AGP.

Overall, higher relative abundances of regulatory, membrane-associated, and metabolic functional categories were observed in CCF1.0 compared with AGP, while the core functional profile remained largely conserved. However, it should be noted that functional predictions generated by PICRUSt2 are inferential and do not represent direct measurements.

## 4. Discussion

Coffee cherry pulp (CCP) is an abundant by-product of coffee processing that contains dietary fiber and diverse bioactive compounds, including polyphenols and caffeine, with recognized antioxidant potential [1,4,13]. Increasing attention has been directed toward the valorization of coffee by-products within circular economy frameworks [2,6]. Microbial fermentation enhance the bioavailability of phenolic compounds and reduce antinutritional factors, thereby improving the functional value of plant-derived feed ingredients [11,25]. In the present study, CCP fermented with *Kluyveromyces marxianus* ST5 was evaluated as a dietary supplement in Thai native chickens, extending previous in vitro findings that demonstrated enhanced antioxidant activity and peptide content after fermentation [10]. Dietary supplementation with 1.0 g/kg CCF resulted in the highest final body weight and ADG, together with the lowest FCR, compared with other groups. These results suggest that moderate inclusion of fermented CCP may enhance feed efficiency without increasing feed intake. The improved FCR may reflect enhanced nutrient utilization rather than simple stimulation of appetite. Comparable improvements in performance have been reported with coffee-derived supplements in broilers [12,17]. Bioactive compounds present in coffee pulp, particularly phenolics, exert antioxidant and antimicrobial effects that can influence gut microbial balance [10,26]. A more stable intestinal environment may enhance digestive efficiency and nutrient absorption, consistent with the observed enrichment of SCFA-associated genera, thereby contributing to improved growth performance. However, increasing the inclusion level to 2.0 g/kg did not yield additional performance gains. High concentrations of polyphenols can interact with dietary proteins and minerals [27], which may partly explain the absence of a linear dose response. The data therefore suggest that 1.0 g/kg represents a biologically appropriate inclusion level under the present conditions.

CCF supplementation significantly reduced serum triglycerides at 1.0 and 2.0 g/kg, while most liver and kidney function indicators remained within normal ranges. Chlorogenic acid, a major phenolic compound in coffee pulp [1,26], modulate lipid metabolism, potentially through effects on hepatic lipid synthesis and triacyl glyceride synthesis [28,29]. Similar hypolipidemic effects of coffee by-products have been documented in poultry and rodent models [17,30]. Variations in ALP activity observed among treatments may reflect physiological processes associated with growth and mineral metabolism in poultry rather than pathological conditions. In growing chickens, ALP activity is closely associated with bone formation and intestinal activity, and moderate fluctuations are commonly reported without indicating liver dysfunction when other hepatic indicators such as AST and ALT remain within normal ranges [31]. Therefore, the observed differences in ALP among treatments may represent normal metabolic variation rather than adverse physiological effects. The higher globulin concentration observed in the CCF2.0 group may suggest modulation of immune-related protein synthesis. Globulins include immunoglobulins and other immune-associated proteins that play important roles in host defense. Plant-derived feed additives rich in polyphenols and antioxidant compounds have been reported to influence immune responses and may increase circulating globulin concentrations in poultry [32]. In the same way, the absence of significant changes in AST, ALT, creatinine, and total protein indicates that CCF supplementation did not adversely affect hepatic or renal function. These findings support the safety of fermented CCP as a feed additive at the tested levels.

CCF supplementation improved certain functional meat quality parameters, particularly by reducing cooking loss and increasing thigh muscle crude protein content at 0.5 and 1.0 g/kg. Improvements in water-holding capacity are frequently associated with enhanced oxidative stability of muscle proteins. Dietary antioxidants improve meat oxidative stability and quality parameters in broilers [33,34]. Because postmortem pH was unaffected, the reduction in cooking loss likely reflects structural preservation of muscle proteins rather than altered glycolysis [35]. In addition, the increased crude protein content in thigh muscle suggests enhanced protein deposition or altered nutrient partitioning under moderate CCF supplementation. Fermentation of coffee cherry pulp can generate bioactive metabolites and increase the bioaccessibility of polyphenolic compounds [10], which could enhance antioxidant status and improve protein metabolism in muscle tissue [36]. Such effects may promote muscle protein accretion while maintaining structural integrity of myofibrillar proteins, thereby contributing to improved water-holding capacity [37,38]. Polyphenol-rich feed additives have been associated with improved nitrogen retention and modulation of muscle metabolism [39]. The absence of differences in shear force indicates that enhanced protein deposition did not compromise tenderness. The increase in L* value observed in the CCF2.0 group may reflect improved oxidative stability of muscle pigments, as antioxidants can reduce myoglobin oxidation and preserve meat brightness [33,40]. However, redness and yellowness were not significantly altered, indicating selective effects on meat appearance.

Alpha diversity indices were maintained across treatments, indicating that CCF supplementation did not disrupt overall microbial richness. Although beta diversity did not reveal statistically significant separation, directional shifts were observed, suggesting subtle modulation of community structure. Enrichment of genera such as UCG-005 and *Subdoligranulum* was noted in CCF groups These taxa are commonly linked to the production of short-chain fatty acids [41,42,43]. SCFAs play key roles in intestinal epithelial integrity, energy metabolism, and immune modulation. An increase in SCFA-producing taxa may improve intestinal barrier function and nutrient absorption, thereby contributing to improved growth and feed efficiency. Similar microbiota-mediated effects have been reported for plant-derived functional additives in poultry [12,17,30].

Functional prediction based on COG and MetaCyc pathway profiling indicated that the overall functional framework of the cecal microbiota was largely conserved across dietary treatments. This stability is consistent with the maintained alpha diversity observed in the present study and suggests that fermented CCP did not induce functional disruption of the microbial ecosystem. Despite this conserved core, relative differences were observed in specific regulatory and metabolic categories in CCF-supplemented groups. In particular, CCF1.0 and CCF2.0 showed higher relative abundance of transcriptional regulators (e.g., LysR, XRE, MarR families) and signal transduction-related functions compared with AGP. Regulatory proteins of these families participate in bacterial adaptive responses to environmental and nutritional changes, including oxidative stress and substrate availability. The higher relative abundance of these regulatory categories may be predicted with microbial adaptation to dietary bioactive compounds, particularly phenolics present in coffee by-products [1,44,45]. Membrane-associated and transport-related functions were also relatively enriched in CCF groups. ABC (ATP-Binding Cassette) transporters and efflux systems are often involved in nutrient uptake, metabolite exchange, and detoxification processes. Polyphenolic compounds can exert selective pressure on microbial communities, leading to adaptive shifts in membrane transport systems [45]. Such predicted differences may reflect inferred shifts in microbial metabolic potential rather than major taxonomic restructuring.

At the pathway level, CCF1.0 demonstrated modest increases in central carbohydrate metabolism, including glycolysis and the non-oxidative pentose phosphate pathway. Predicted enrichment of carbohydrate metabolism pathways may reflect increased fermentative potential of the microbial community. Fermentation end-products such as short-chain fatty acids (SCFAs) play essential roles in intestinal epithelial integrity and host energy metabolism. Coffee-derived substrates have previously been reported to influence gut microbial fermentation patterns [15,30], supporting the plausibility of this functional shift. Predicted increases in branched-chain amino acid biosynthesis pathways were also observed in CCF1.0. Microbial amino acid synthesis can contribute to luminal amino acid availability and nitrogen recycling in poultry [42]. Although the contribution of microbial amino acid production to host protein accretion remains difficult to quantify, modulation of these pathways aligns with the increased crude protein content observed in thigh muscle at moderate inclusion levels. It should be noted that PICRUSt-based functional prediction infers metabolic potential from 16S rRNA gene profiles rather than directly measuring gene abundance or expression. While this approach provides valuable insight into community-level trends, it does not replace whole-metagenome sequencing or metabolomic validation [22]. Therefore, the functional shifts described here should be interpreted as indicative rather than definitive evidence of microbial metabolic activity.

Overall, the findings suggest that fermented CCP exerts its beneficial effects through a coordinated gut–metabolism–muscle axis. The findings support a conceptual gut–metabolism–muscle axis, in which fermented CCP supplementation was associated with shifts in microbial composition and predicted metabolic pathways, alongside improvements in feed efficiency and muscle protein content. Therefore, fermented CCP represents a promising natural alternative to antibiotic growth promoters for improving performance and meat quality in Thai native chickens. From a practical perspective, the optimal supplementation level identified in this study (1.0 g/kg diet) represents a relatively low inclusion rate, which may be feasible for incorporation into commercial poultry diets without substantially altering feed formulation. The optimal response observed at 1.0 g/kg indicates that moderate supplementation balances antioxidant benefits and nutrient availability without inducing excessive polyphenol–protein interactions. Therefore, fermented CCP represents a promising natural alternative to antibiotic growth promoters for improving performance and meat quality in Thai native chickens.

## 5. Conclusions

Fermented coffee cherry pulp (CCF) at 1.0 g/kg improved growth performance, feed efficiency, triglyceride, and selected meat quality traits in Thai native chickens without adverse health effects. CCF supplementation maintained overall microbial diversity while inducing directional compositional shifts, including enrichment of short-chain fatty acid (SCFA)-associated genera. Functional prediction analysis suggested modulation of microbial metabolic potential, particularly in pathways related to carbohydrate and amino acid metabolism, although the core functional structure remained conserved. These findings suggest that moderate CCF supplementation represents a promising natural alternative to antibiotic growth promoters in native chicken production.

## Figures and Tables

**Table 1:** Ingredients and nutrient composition of Thai native chicken starter and finisher diets.

Item	Age 0–6 Week	Age 7–14 Week
**Ingredients**		
Corn meal	700.00	575.00
Rice bran	0.00	75.00
Full fat soybean meal	0.00	25.00
Soybean meal 44%	205.00	192.50
Meat meal 50%	25.00	25.00
Limestone	10.00	25.00
Calcium carbonate	0.00	47.40
Monopotassium phosphate 22%	10.50	17.50
Premix ^1^	2.50	2.50
Methionine	0.90	1.50
Toxin binder	1.00	0.50
Salt	0.00	2.00
Multi protein plus 68%	45.00	11.00
Phytase	0.10	0.10
	**1000.00**	**1000.00**
**Nutrient composition (calculated)**		
Crude protein	24.22	15.00
Moisture	12.23	9.78
Ash	6.79	11.91
Crude fiber	4.56	3.82
Crude fat	5.15	4.59
Gross energy (Cal/g)	2964.92	3581.65

^1^ Mineral premix supplies per kg of diet: vitamin A 20,000,000 IU, vitamin D3 4,000,000 IU, vitamin E 11,000 IU, vitamin K3 4.00 g, vitamin B1 5.00 g, vitamin B2 10.00 g, vitamin B6 5.00 g, vitamin B12 0.02 g, vitamin C 15.00 g, pantothenic acid 15.00 g, folic acid 3.00 g, nicotinic acid 40.00 g, biotin 0.20 g, magnesium 100.00 g, potassium 90.00 g, sodium 100.00 g, and feed additive 25.30 g.

**Table 2:** Effects of different levels of dietary fermented coffee cherry pulp supplementation on the growth performance of Thai native chickens.

Items ^a^	CON	AGP	CCF0.5	CCF1.0	CCF2.0	SEM	*p*-Value
Initial weight (g)	34.17	34.87	33.4	35.27	35.30	0.598	0.844
Final weight (g)	2087.80 ^b^	2216.90 ^ab^	2128.70 ^b^	2335.30 ^a^	2168.80 ^b^	24.275	0.001
ADG (g/d)	24.45 ^b^	25.98 ^ab^	24.94 ^b^	27.38 ^a^	25.40 ^b^	0.285	0.009
ADFI (g/bird/d)	88.51 ^c^	92.53 ^a^	90.80 ^b^	83.43 ^d^	91.13 ^ab^	0.508	0.001
FCR	3.63 ^b^	3.60 ^b^	3.65 ^b^	3.05 ^a^	3.59 ^b^	0.048	0.001

^a–d^ Means within a row with different superscripts are different at *p* < 0.05. ADG, average daily gain; ADFI, average daily feed intake; FCR, feed conversion ratio. SEM, standard error of the mean. CON = control group; AGP = birds supplemented with antibiotic; CCF0.5 = birds supplemented with 0.5 g/kg fermented coffee cherry pulp; CCF1.0 = birds supplemented with 1.0 g/kg fermented coffee cherry pulp; CCF2.0 = birds supplemented with 2.0 g/kg fermented coffee cherry pulp.

**Table 3:** Effects of different levels of dietary fermented coffee cherry pulp supplementation on the serum biochemical parameters and lipid profile of Thai native chickens.

Items	Basal	AGP	CCF0.5	CCF1.0	CCF2.0	SEM	*p*-Value
**Serum biochemistry**							
BUN (mg/dL)	2.00 ^a^	1.16 ^b^	1.26 ^b^	1.67 ^ab^	1.67 ^ab^	0.089	0.011
Creatinine (mg/dL)	0.33	0.33	0.32	0.40	0.34	0.010	0.157
AST (U/L)	276.80	217.33	226.00	265.67	229.00	7.947	0.050
ALT (U/L)	4.20	1.67	2.00	3.33	3.00	0.312	0.057
ALP (U/L)	1062.80 ^a^	821.33 ^b^	821.33 ^b^	1136.67 ^a^	850.33 ^b^	35.774	0.002
Total protein (g/dL)	4.24	4.10	3.70	4.67	4.10	0.122	0.161
Albumin (g/dL)	1.74	1.60	1.50	1.77	1.60	0.051	0.480
Globulin (g/dL)	2.50 ^b^	2.50 ^b^	2.20 ^b^	2.90 ^b^	4.47 ^a^	0.263	0.036
**Lipid profile**							
Total cholesterol (mg/dL)	156.40	153.33	143.00	183.33	165.67	5.432	0.178
Triglyceride (mg/dL)	39.00 ^ab^	45.33 ^a^	40.67 ^ab^	30.67 ^bc^	25.33 ^c^	2.167	0.014
HDL (mg/dL)	120.78	132.63	116.77	146.90	133.57	4.127	0.150
LDL (mg/dL)	35.02	40.60	40.30	31.40	26.47	1.939	0.091

^a–c^ Means within a row with different superscripts are different at *p* < 0.05. BUN: blood urea nitrogen; AST: aspartate aminotransferase; ALT: alanine transaminase; ALP: alkaline phosphatase; HDL: high-density lipoprotein; LDL: low-density lipoprotein. SEM: standard error of the mean. Basal: the control diet group; AGP: birds supplemented with antibiotic; CCF0.5, 1.0 and 2.0: birds supplemented with 0.5, 1.0 and 2.0 g/kg fermented coffee cherry pulp.

**Table 4:** Effects of different levels of dietary coffee cherry pulp fermented supplementation on meat quality and muscle chemical composition of Thai native chickens.

Items	Basal	AGP	CCF0.5	CCF1.0	CCF2.0	SEM	*p*-Value
pH of breast muscle							
45 min	5.74	5.82	5.82	5.79	5.82	0.020	0.681
24 h	5.34	5.25	5.75	5.73	5.65	0.079	0.105
Decline in pH after 24 h	0.40	0.57	0.07	0.06	0.17	0.085	0.238
Meat color (Breast) ^1^						-	-
L* (lightness)	50.78 ^b^	52.59 ^b^	52.34 ^b^	52.39 ^b^	58.70 ^a^	0.892	0.015
a* (redness)	−1.35	−1.62	0.19	0.03	1.79	0.450	0.085
b* (yellowness)	12.17	11.98	12.66	13.83	17.09	0.755	0.169
Water holding capacity, %						-	-
Drip loss 24 h	2.69	2.53	2.06	1.64	0.90	0.270	0.218
Cooking loss	25.63 ^a^	25.37 ^b^	25.35 ^b^	25.36 ^b^	25.41 ^b^	0.026	0.001
Shear force (Kg)	3.32	3.32	3.31	3.31	3.32	0.021	0.918
Chemical composition of breast muscle, %							
% DM	93.96	93.37	93.58	92.49	93.69	0.226	0.325
% Moisture	6.04	6.63	6.42	7.51	6.31	0.236	0.235
% Ash	6.01	4.66	4.59	4.61	4.58	0.221	0.150
% EE	2.70	2.22	2.48	2.63	2.59	0.081	0.384
% CP	89.28	88.88	87.00	86.53	88.31	0.387	0.078
Chemical composition of thigh muscle, %							
% DM	94.59 ^c^	95.81 ^a^	95.53 ^a^	95.69 ^a^	95.01 ^b^	0.128	0.001
% Moisture	5.40 ^a^	4.18 ^c^	4.46 ^c^	4.30 ^c^	4.98 ^b^	0.124	0.001
% Ash	4.76	4.52	4.58	4.45	4.57	0.005	0.474
% EE	13.90	13.21	11.68	11.82	12.18	0.600	0.786
% CP	79.03 ^c^	77.53 ^c^	82.37 ^a^	80.78 ^ab^	80.43 ^ab^	0.491	0.002

^a–c^ Means within a row with different superscripts are different at *p* < 0.05. ^1^ L*: lightness; a*: redness; b*: yellowness. SEM: standard error of the mean. Basal: the control diet group; AGP: birds supplemented with antibiotic; CCF0.5, 1.0 and 2.0: birds supplemented with 0.5, 1.0 and 2.0 g/kg fermented coffee cherry pulp.

## Data Availability

Data are available on request due to restrictions, e.g., privacy or ethical restrictions. The data presented in this study are available on request from the corresponding author. The data are not publicly available due to the law of the Ministry of Higher Education, Science, Research, and Innovation.

## Supplementary Materials

The following supporting information can be downloaded at: https://www.mdpi.com/article/10.3390/ani16060965/s1, Table S1: Predicted relative abundances and functional annotation of Cluster of Orthologous Groups (COG) categories across dietary treatments based on PICRUSt analysis; Table S2: Relative abundance of MetaCyc pathways identified by PICRUSt functional prediction across treatments.
