# Supplementary material for: Influence of Dietary Fermented Coffee Cherry Pulp on Growth Performance, Meat Quality, and Cecal Microbiota in Thai Native Chickens
Source: Animals (Basel). 2026 Mar 19;16(6):965. doi: 10.3390/ani16060965 (PMC13023244; doi:10.3390/ani16060965)
Supplement: Supplementary file 1 [file animals-16-00965-s001.zip › animals-4202252-supplementary.pdf]

Supplementary Table

Supplementary Table S1. Predicted relative abundances and functional annotation of Cluster of Orthologous Groups (COG) categories across dietary treatments based on PICRUSt analysis.

Function	Functional description	Basal	AGP	CCF0.5	CCF1.0	CCF2.0
**COG0436**	Aspartate/methionine/tyrosine aminotransferase	408,327.55	392,786.14	389,991.14	398,362.92	400,206.43
**COG0438**	Glycosyltransferase involved in cell wall bisynthesis	685,607.41	708,519.49	691,056.94	684,256.03	682,588.38
**COG0451**	Nucleoside-diphosphate-sugar epimerase	341,340.29	351,346.54	340,877.36	339,444.27	343,227.35
**COG0456**	Ribosomal protein S18 acetylase RimI and related acetyltransferases	399,428.74	377,835.98	389,257.07	384,428.39	380,864.39
**COG0463**	Glycosyltransferase involved in cell wall bisynthesis	464,326.60	446,459.24	441,494.82	433,659.57	433,844.28
**COG0488**	ATPase components of ABC transporters with duplicated ATPase domains	401,889.87	388,895.99	398,606.80	396,393.77	395,637.51
**COG0524**	Sugar or nucleoside kinase, ribokinase family	361,230.59	344,945.84	352,719.83	362,280.26	354,444.71
**COG0534**	Na+-driven multidrug efflux pump	896,932.46	841,298.96	871,212.48	888,303.22	887,908.16
**COG0564**	Pseudouridylate synthase, 23S rRNA- or tRNA-specific	344,830.81	327,693.66	328,532.55	332,516.23	335,825.51
**COG0583**	DNA-binding transcriptional regulator, LysR family	687,776.77	632,648.48	627,381.86	629,686.73	625,353.41
**COG0642**	Signal transduction histidine kinase	1,045,423.78	967,327.03	987,377.81	1,006,419.91	1,006,468.69
**COG0664**	cAMP-binding domain of CRP or a regulatory subunit of cAMP-dependent protein kinases	413,925.59	378,280.10	397,476.57	421,980.18	431,901.19
**COG0745**	DNA-binding response regulator, OmpR family, contains REC and winged-helix (wHTH) domain	983,677.87	914,756.89	919,756.98	936,161.31	937,284.56
**COG0778**	Nitroreductase	409,859.16	399,140.66	411,162.12	419,090.75	414,238.27
**COG0789**	DNA-binding transcriptional regulator, MerR family	469,271.09	435,240.75	443,743.78	436,738.38	434,598.38
**COG1028**	NAD(P)-dependent dehydrogenase, short-chain alcohol dehydrogenase family	692,449.18	658,148.01	654,617.28	662,730.96	662,152.38
**COG1131**	ABC-type multidrug transport system, ATPase component	874,471.17	818,945.57	820,398.93	824,202.87	828,787.09

Function	Functional description	Basal	AGP	CCF0.5	CCF1.0	CCF2.0
**COG1132**	ABC-type multidrug transport system, ATPase and permease component	839,833.66	774,191.08	771,384.98	778,908.54	778,102.37
**COG1136**	ABC-type lipoprotein export system, ATPase component	690,400.62	659,558.02	661,810.69	657,062.77	655,066.72
**COG1191**	DNA-directed RNA polymerase specialized sigma subunit	422,001.01	379,745.23	381,246.41	395,249.47	395,937.44
**COG1309**	DNA-binding transcriptional regulator, AcrR family	810,487.16	747,420.47	755,128.18	760,397.66	773,902.02
**COG1396**	Transcriptional regulator, contains XRE-family HTH domain	345,788.54	305,029.97	306,341.13	320,593.62	320,969.59
**COG1476**	DNA-binding transcriptional regulator, XRE-family HTH domain	708,131.83	636,205.42	648,894.36	669,633.83	678,068.21
**COG1595**	DNA-directed RNA polymerase specialized sigma subunit, sigma24 family	925,067.73	882,209.85	916,740.63	944,321.46	937,112.18
**COG1609**	DNA-binding transcriptional regulator, LacI/PurR family	522,842.45	471,154.62	486,873.62	476,220.22	480,853.65
**COG1653**	ABC-type glycerol-3-phosphate transport system, periplasmic component	450,770.08	396,755.45	409,660.55	401,950.07	408,258.31
**COG1670**	Protein N-acetyltransferase, RimJ/RimL family	382,159.32	366,914.29	372,513.41	372,278.30	374,920.49
**COG1846**	DNA-binding transcriptional regulator, MarR family	653,828.16	595,601.64	603,328.40	599,616.21	605,925.76
**COG1961**	Site-specific DNA recombinase related to the DNA invertase Pin	679,494.84	589,767.22	621,362.85	659,554.13	669,504.37
**COG2199**	GGDEF domain, diguanylate cyclase (c-di-GMP synthetase) or its enzymatically inactive variants	463,326.78	408,551.60	418,703.91	405,097.03	412,294.13
**COG2207**	AraC-type DNA-binding domain and AraC-containing proteins	846,640.08	784,560.59	818,447.18	822,617.49	826,335.44
**COG2244**	Membrane protein involved in the export of O-antigen and teichoic acid	381,385.34	359,745.59	385,653.64	385,062.26	386,808.80
**COG2814**	Predicted arabinose efflux permease, MFS family	362,565.73	367,573.22	372,315.51	381,128.60	384,000.36
**COG4624**	Iron only hydrogenase large subunit, C-terminal domain	343,855.02	308,176.94	318,666.86	325,852.36	331,568.97
**COG4974**	Site-specific recombinase XerD	875,127.51	825,998.36	860,518.60	882,749.61	878,434.85

CON = the control group; AGPs = birds supplemented with 0.25 mg/kg Antibiotic; CCF0.5 = birds supplemented with 0.5 g/kg fermented coffee cherry pulp; CCF1.0 = birds supplemented with 1.0 g/kg fermented coffee cherry pulp; CCF2.0 = birds supplemented with 2.0 g/kg fermented coffee cherry pulp.

Supplementary Table S2. Relative abundance of MetaCyc pathways identified by PICRUSt functional prediction across treatments.

Pathway ID	Functional description	Basal	AGP	CCF0.5	CCF1.0	CCF2.0
**ANAGLYCOLYSIS-PWY**	glycolysis III (from glucose)	127,812.05	125,688.55	126,705.23	128,054.98	127,292.74
**ARO-PWY**	chorismate biosynthesis I	121,099.66	117,811.03	116,750.39	118,182.41	117,486.20
**BRANCHED-CHAIN-AA-SYN-PWY**	superpathway of branched amino acid biosynthesis	131,376.24	128,130.29	127,715.02	128,748.07	128,607.16
**CALVIN-PWY**	Calvin-Benson-Bassham cycle	119,263.42	117,206.87	115,547.68	115,045.15	113,818.12
**COMPLETE-ARO-PWY**	superpathway of aromatic amino acid biosynthesis	126,651.45	122,836.04	121,909.42	123,585.77	122,952.14
**GLYCOGENSYNTH-PWY**	glycogen biosynthesis I (from ADP-D-Glucose)	122,846.39	119,013.52	118,518.48	120,422.63	120,052.05
**ILEUSYN-PWY**	L-isoleucine biosynthesis I (from threonine)	134,987.25	130,381.24	130,804.83	132,338.47	132,254.16
**NONOXIPENT-PWY**	pentose phosphate pathway (non-oxidative branch)	157,448.42	152,558.63	152,434.50	153,864.38	152,566.97
**PHOSLIPSYN-PWY**	superpathway of phospholipid biosynthesis I (bacteria)	124,337.83	120,139.30	118,504.33	119,939.88	120,073.21
**PWY-2942**	L-lysine biosynthesis III	125,516.67	123,285.47	123,200.49	123,535.38	123,960.71
**PWY-3001**	superpathway of L-isoleucine biosynthesis I	127,480.51	124,710.06	123,888.07	125,791.42	125,377.82
**PWY-5097**	L-lysine biosynthesis VI	123,977.89	121,206.82	121,508.94	121,721.48	122,002.61
**PWY-5100**	pyruvate fermentation to acetate and lactate II	118,995.10	115,985.08	112,599.72	116,837.10	116,106.08
**PWY-5101**	L-isoleucine biosynthesis II	146,496.23	141,725.78	142,156.72	143,792.69	143,736.81
**PWY-5103**	L-isoleucine biosynthesis III	127,965.15	124,546.39	124,156.06	125,580.41	125,544.75
**PWY-5104**	L-isoleucine biosynthesis IV	139,670.38	135,402.95	135,544.81	137,859.97	137,061.84
**PWY-5667**	CDP-diacylglycerol biosynthesis I	132,475.22	128,703.83	127,996.39	129,486.75	129,335.16
**PWY-5686**	UMP biosynthesis	126,663.72	123,331.62	122,707.25	123,749.04	123,576.86
**PWY-5973**	cis-vaccenate biosynthesis	131,314.81	127,798.07	127,246.53	129,350.70	129,872.16
**PWY-6121**	5-aminoimidazole ribonucleotide biosynthesis I	122,019.93	121,279.93	120,365.28	121,019.53	120,463.86

Pathway ID	Functional description	Basal	AGP	CCF0.5	CCF1.0	CCF2.0
**PWY-6122**	5-aminoimidazole ribonucleotide biosynthesis II	122,233.78	120,934.92	119,217.46	119,801.32	119,443.04
**PWY-6126**	superpathway of adenosine nucleotides de novo biosynthesis II	123,786.22	121,770.15	120,829.50	123,317.58	124,238.12
**PWY-6277**	superpathway of 5-aminoimidazole ribonucleotide biosynthesis	122,233.78	120,934.92	119,217.46	119,801.32	119,443.04
**PWY-6386**	UDP-N-acetylmuramoyl-pentapeptide biosynthesis II (lysine-containing)	120,846.09	118,222.58	117,432.43	118,679.86	119,068.32
**PWY-6387**	UDP-N-acetylmuramoyl-pentapeptide biosynthesis I (meso-diaminopimelate containing)	119,327.40	116,958.34	115,817.79	117,064.94	117,325.22
**PWY-7111**	pyruvate fermentation to isobutanol (engineered)	147,198.98	140,567.85	139,018.85	139,735.58	140,527.71
**PWY-7208**	superpathway of pyrimidine nucleobases salvage	136,832.84	134,552.75	132,694.77	133,724.23	133,795.80
**PWY-7219**	adenosine ribonucleotides de novo biosynthesis	134,074.69	131,690.23	130,111.23	131,644.02	131,837.33
**PWY-7229**	superpathway of adenosine nucleotides de novo biosynthesis I	128,132.54	125,764.71	124,770.55	126,830.51	127,476.72
**PWY-7663**	gondoate biosynthesis (anaerobic)	135,923.37	132,452.75	131,626.86	134,088.13	134,576.71
**PWY0-1319**	CDP-diacylglycerol biosynthesis II	132,475.22	128,703.83	127,996.39	129,486.75	129,335.16
**PWY4FS-7**	phosphatidylglycerol biosynthesis I (plastidic)	119,520.11	115,071.28	113,000.39	114,350.48	114,628.81
**PWY4FS-8**	phosphatidylglycerol biosynthesis II (non-plastidic)	119,520.11	115,071.28	113,000.39	114,350.48	114,628.81
**THRESYN-PWY**	superpathway of L-threonine biosynthesis	124,158.61	122,041.50	120,812.48	123,050.51	122,434.14
**VALSYN-PWY**	L-valine biosynthesis	134,987.25	130,381.24	130,804.83	132,338.47	132,254.16

CON = the control group; AGPs = birds supplemented with 0.25 mg/kg Antibiotic; CCF0.5 = birds supplemented with 0.5 g/kg fermented coffee cherry pulp; CCF1.0 = birds supplemented with 1.0 g/kg fermented coffee cherry pulp; CCF2.0 = birds supplemented with 2.0 g/kg fermented coffee cherry pulp.
